# Preparation of Single-Cell Suspensions from Mouse Spleen with the gentleMACS Dissociator

**DOI:** 10.3791/1029

**Published:** 2008-12-11

**Authors:** Melanie Jungblut, Karen Oeltze, Irene Zehnter, Doris Hasselmann, Andreas Bosio

**Affiliations:** Miltenyi Biotec,GmbH

## Abstract

Single-cell suspensions are a prerequisite for experiments in cell separation, cell analysis and cell culture.  To avoid tedious and often painful manual dissociations the gentleMACS Dissociator allows one to dissociate tissue very efficiently under controlled and reproducible conditions.  The gentleMACS Dissociator can optimally dissociate mouse spleen, combining timesaving and standardization with user-safety. This video describes how to dissociate mouse spleens using the gentleMACS Dissociator, an automated bench-top device that can mechanically disrupt tissues using special tubes to produce viable cell suspensions. Following dissociation, spleens are filtered, centrifuged, and resuspended for further applications.

**Figure:**
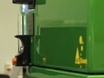


## Protocol

### Introduction to Equipment

Tissue is gently dissociated, using violet C Tubes into viable single-cell suspensions.
The PBS/BSA EDTA buffer is used to dissociate the cells.  The buffer is prepared by adding 1 part of BSA Stock solution to 20 parts of autoMACS Rinsing Solution containing EDTA.
The MACS Pre-Separation Filters are used so that the dissociated tissue samples are free of all cell clumps, promoting successful MACS separation and subsequent analysis.

### Dissociating the spleen cells

Whole spleens are disscociated into single-cell suspensions of splenocytes using the gentleMACS Dissociator.
To begin the procedure, load the C Tube with a volume of buffer according to the mass of the spleens.  A healthy spleen of a young adult female BALB/c mouse weighs about 80-120 mg. Every 1 to 2 spleens of this mass require 3 mL of buffer. Spleens from older animals or animals with infections are often larger and must be weighed in buffer. The buffer volumes must be scaled accordingly. Do not exceed a total buffer volume of 10 mL.
Add the dissected spleen to the C Tube containing 3 ml of buffer.
Make sure that the C Tube cap is tight and attach the C Tube to the dissociator with the cap down.
Power up the dissociator. For 1-2 spleens, select program m_spleen_01. The program will take 56 seconds to complete. If the number of spleens is greater, use program m_spleen_04; this program can be used for up to 720 mg of spleen tissue.
After completion of the program the native spleen tissue is dissociated into a splenocyte suspension.
Spin down the whole cell suspension at 300xg at room temperature for about 30 sec. This will ensure that rotor and stator are freed of all cells which are collected at the bottom of the C Tube.
Remove the sample through the septum-sealed opening in the C Tube cap using a 1000 microliter pipetter.

### Filtration step

To begin the filtration step, apply the dissociated tissue to the Pre-Separation Filter or a cell strainer with 30 µm pore size.  The strainer should fit a 15 mL tube for 1-2 spleens or a 50 mL tube for more than 2 spleens.
Let the cell suspension pass through. Then wash the filter by applying 5 mL of buffer.
Now, centrifuge the tube with the cell suspension at 300xg, at room temperature for 10 minutes to collect all splenocytes in a spleen cell pellet.
Now, that we have a pellet of dissociated splenocytes, we must aspirate the supernatant and resuspend the cell pellet in an appropriate amount of buffer.

Spleen dissociation using the gentleMACS Dissociator will yield a high percentage of viable splenocytes, which is demonstrated by flow cytometry using the MACSQuant Analyzer.  Dead cells are stained by propidium iodide. In the example provided, the forward side scatter dot plot shows the prepared splenocytes.

## Disclosures

All protocols and data sheets are available at  <a href="http://www.miltenyibiotec.com">http://www.miltenyibiotec.com.</a>.

## Discussion

To avoid tedious and often painful manual dissociations, the gentleMACS Dissociator allows one to dissociate tissue very efficiently under controlled and reproducible conditions. The tissue dissociation procedure is gentle to cells and yields cell suspensions with a high viability rate. The gentleMACS Dissociator in combination with C Tubes is designed for the preparation of single-cell suspensions from various tissues. The instrument is equipped with a number of pre-set programs to dissociate mouse spleen, liver, lung, or neural tissue. The range of programs and protocols will be continuously expanded to include additional tissues.

